# Limitations to causal inference in observational studies of PFO closure

**DOI:** 10.1093/esj/23969873251368726

**Published:** 2026-01-01

**Authors:** Iyas Daghlas

**Affiliations:** UCSF Weill Institute for Neurosciences, Department of Neurology, University of California San Francisco, San Francisco, CA, USA

**Keywords:** PFO, causal inference, epidemiology%

To the editor,

Wang et al.^1^ are to be commended for investigating a timely and clinically relevant topic. While the authors present encouraging results, several methodological limitations merit consideration before drawing causal conclusions.

First, there were significant baseline differences between the PFOC and non-PFOC groups with respect to the distribution of ischemic stroke risk factors, raising concerns for bias due to unmeasured confounding. Although adjusted analyses included the RoPE score as a covariate, this is a less efficient approach compared to adjusting for the individual components of the score.

Second, the groups differed substantially with respect to markers predictive of PFO-attributable thromboembolic risk, including shunt size, presence of atrial septal aneurysm, and burden of high-intensity transient signals. These measures were not included in multivariable models, rendering the study at risk for confounding by indication. As a result, the study may be better conceptualized as comparing PFO closure in patients with PFO-attributable stroke to medical therapy in patients with incidental PFO. This estimand is neither readily interpretable nor comparable to that from randomized controlled trials. Application of a target trial emulation framework could have mitigated this concern and improved causal interpretability.^2^

Third, the potential for immortal time bias raises concern.^3^ The interval between the index stroke and PFO closure introduces a window during which patients assigned to the PFOC group were “immortal” and could not have experienced a recurrent stroke before receiving the intervention. It is unclear from the provided methodology whether the time-to-event analyses initiated follow-up from the date of PFOC or the index event. Inclusion of Kaplan-Meier curves would have helped with visual assessment for this potential bias.

Fourth, it is not apparent why the duration of follow-up differed significantly between the PFOC and non-PFOC groups; correlation of differential follow-up with clinical risk factors could introduce bias into the survival analysis through informative censoring.

Fifth, it is unclear whether the reduction in ischemic stroke risk is attributable to prolonged treatment with dual antiplatelet therapy, or to PFOC. Sixth, no operational definition of recurrent ischemic stroke was provided, making it difficult to evaluate the specificity of outcome ascertainment. Moreover, it is unclear whether adjudicators of stroke events were blinded to treatment assignment. Finally, as the authors acknowledge, they employed 24-h Holter monitoring to exclude atrial fibrillation. In an elderly cohort, this short duration of cardiac monitoring is insufficient to exclude paroxysmal AF, potentially misclassifying patients with cardioembolic stroke as cryptogenic.

Considering these limitations, the conclusion that “PFO closure is safe and effective in elderly patients with CS” appears overstated. Future studies examining this association should implement the target trial emulation framework, carefully account for selection bias, and take measures to avoid immortal time bias. Ultimately, high-quality randomized controlled trials are required prior to modification of clinical practice.

Sincerely,


Iyas Daghlas, MD

